# Prediction of Drug-Induced Hepatotoxicity Using Long-Term Stable Primary Hepatic 3D Spheroid Cultures in Chemically Defined Conditions

**DOI:** 10.1093/toxsci/kfy058

**Published:** 2018-03-24

**Authors:** Sabine U Vorrink, Yitian Zhou, Magnus Ingelman-Sundberg, Volker M Lauschke

**Affiliations:** Section of Pharmacogenetics, Department of Physiology and Pharmacology, Karolinska Institutet, Stockholm SE-171 77, Sweden

**Keywords:** liver, toxicity screen, drug development, species comparison, *in vitro*

## Abstract

High failure rates of drug candidates in the clinics, restricted-use warnings as well as withdrawals of drugs in postmarketing stages are of substantial concern for the pharmaceutical industry and drug-induced liver injury (DILI) constitutes one of the most frequent reasons for such safety failures. Importantly, as DILI cannot be accurately predicted using animal models, animal safety tests are commonly complemented with assessments in human *in vitro* systems. 3D spheroid cultures of primary human hepatocytes in chemically defined conditions, hereafter termed CD-spheroids, have recently emerged as a microphysiological model system in which hepatocytes retain their molecular phenotypes and hepatic functions for multiple weeks in culture. However, their predictive power for the detection of hepatotoxic liabilities has not been systematically assessed. Therefore, we here evaluated the hepatotoxicity of 123 drugs with or without direct implication in clinical DILI events. Importantly, using ATP quantifications as the single endpoint, the model accurately distinguished between hepatotoxic and nontoxic structural analogues and exceeded both sensitivity and specificity of all previously published *in vitro* assays at substantially lower exposure levels, successfully detecting 69% of all hepatotoxic compounds without producing any false positive results (100% specificity). Furthermore, the platform supports the culture of spheroids of primary hepatocytes from preclinical animal models, thereby allowing the identification of animal-specific toxicity events. We anticipate that CD-spheroids represent a powerful and versatile tool in drug discovery and preclinical drug development that can reliably flag hepatotoxic drug candidates and provide guidance for the selection of the most suitable animal models.

Adverse drug reactions (ADRs) are of major concern for the pharmaceutical industry (Kaplowitz, 2005; Lauschke *et al.*, 2018). They are the prime reason for the attrition of promising candidate drugs in development (Waring *et al.*, 2015) and provoked the release of safety communications, black box warnings and withdrawals in postmarketing stages for 32% of medications approved by FDA between 2001 and 2010 (Downing *et al.*, 2017). The liver is among the organ systems most commonly affected by ADRs and drug-induced liver injury (DILI) is the most frequent reason for the withdrawal of drugs from the market (Lasser *et al.*, 2002). Importantly, previous analyses indicate that the quality of preclinical safety profiles is in strong anti-correlation with the risk of project closure due to safety issues in clinical stages (Cook *et al.*, 2014), emphasizing that accurate tests for the prediction of DILI in early stages of drug development are of paramount importance to reduce patient morbidity and to lower drug development costs.

Animal safety tests are required by all major regulatory bodies before a compound can proceed into clinical stages. However, structures, isoform compositions, substrate affinities, and catalytic activities of drug metabolizing enzymes differ substantially between animals and humans (Martignoni *et al.*, 2006). As a result, the concordance between human and animal toxicity is poor with nonrodent and rodent models being predictive for 63% and 43% of human toxicity events (Olson *et al.*, 2000). Moreover, while difficult to quantify, false-positive animal-specific toxicity events can lead to the unnecessary early termination of potentially successful candidate drugs.

To ameliorate these problems, animal experiments are commonly complemented with assessments using human *in vitro* systems (Ewart *et al.*, 2017). A variety of high-content compatible testing models have been developed, including platforms based on hepatoma cell lines (O’Brien *et al.*, 2006; Tolosa *et al.*, 2012), hepatocyte-like cells (iHLCs) derived from induced pluripotent stem cells (iPS) (Sirenko *et al.*, 2014) as well as cell lines overexpressing drug metabolizing enzymes (Gustafsson *et al.*, 2014). Although these systems are relatively inexpensive and universally available, their molecular phenotype drastically differs from human *in vivo* liver tissue, which impairs their predictive power. To improve the quality of hepatotoxicity predictions, much work has been conducted in primary human hepatocytes (PHH) in 2D monolayer cultures, which were considered the gold standard *in vitro* model for drug toxicity testing (Gómez-Lechón *et al.*, 2014). However, since PHH rapidly lose their phenotype within hours in these unphysiological conditions the utility of this model is limited to acute toxicity tests (Lauschke *et al.*, 2016b).

To overcome these limitations, a multitude of more long-term stable hepatic culture systems and conditions have been developed, including 2D sandwich cultures, perfused and static micro-fabricated systems as well as 3D spheroid cultures (Lauschke *et al.*, 2016a; Ware and Khetani, 2016). Although encouraging results have been shown for a multitude of systems, comprehensive evaluations of cellular phenotypes and comparative studies across platforms are mostly lacking. Recently, PHH 3D spheroids in chemically defined conditions, hereafter termed CD-spheroids, have been demonstrated to retain their viability, transcriptomic, proteomic and metabolomic phenotypes, as well as their functionality for multiple weeks (Bell *et al.*, 2016, 2017; Vorrink *et al.*, 2017). Furthermore, the system was benchmarked on phenotypic and functional levels against other emerging cell models, including HepaRG cells, iHLCs and PHH cultured in 2D sandwich configuration (Bell *et al.*, 2017, 2018). However, the extent to which the physiological cellular phenotypes of PHH in 3D spheroid culture can be translated into an improved predictive accuracy in the evaluation of hepatotoxic liabilities of drugs and drug candidates remained to be addressed.

Here, we systematically assessed the predictive accuracy of CD-spheroids to detect compounds implicated in human hepatotoxicity using a panel of 123 drugs with and without direct evidence of causative involvement in clinical DILI events. Using quantifications of ATP levels as a single, easily accessible endpoint, the CD-spheroid model achieved 69% sensitivity and 100% specificity. Furthermore, we demonstrate that the model can faithfully distinguish between hepatotoxic and nontoxic structural analogues and allows for analyses of interspecies differences using primary hepatocytes from different animal models with preclinical importance. Combined, our data indicate that CD-spheroids represent a powerful and versatile tool in drug discovery and preclinical drug development that can reliably flag drug candidates with hepatotoxic liabilities in humans.

## MATERIALS AND METHODS

### 

#### 

##### Cell culture

Cryopreserved PHH were purchased from BioreclamationIVT (Brussels, Belgium) and thawed using *InVitro*GRO CP medium or Cryopreserved Hepatocyte Recovery Medium (Thermo Fisher Scientific, USA). Cells were seeded in 96-well ultra-low attachment plates (Corning, USA) in culture medium (Williams’ medium E, supplemented with 2 mM L-glutamine, 100 U/ml penicillin, 100 mg/ml streptomycin, 10 mg/ml insulin, 5.5 mg/ml transferrin, 6.7 ng/ml sodium selenite, and 100 nM dexamethasone) with 10% fetal bovine serum (FBS) as previously described (Bell *et al.*, 2016). Cryopreserved primary animal hepatocytes were obtained from BioreclamationIVT (Gottingen minipig, Wistar rat, rhesus monkey, CD1 mouse) or Lonza (USA; C57BL/6 mouse; hereafter called BL6) and were thawed according to the supplier’s instructions. Animal cells were seeded at 2000 (rhesus monkey) or 1000 cells/well (minipig, rat and mouse) and exposed as described earlier. Cell culture medium and all medium supplements were purchased from Sigma-Aldrich (Sweden) or Life Technologies (Sweden).

##### Hepatotoxicity evaluations

**Figure 1.**
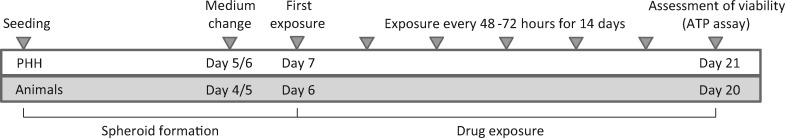
Scheme of the experimental outline. The experimental schedule for all experiments included in this study is shown for both human and animal cells.

This study was part of the Innovative Medicines Initiative Program MIP-DILI and all tested compounds were provided by AstraZeneca Compound Management (Macclesfield, UK) or Sigma Aldrich. The extent of hepatic metabolism was extracted from DrugBank (https://www.drugbank.ca; last accessed January 2018) and the Prescribers’ Digital Reference database (http://www.pdr.net; last accessed January 2018). Acetaminophen was dissolved in culture medium, whereas all other compounds were dissolved in dimethyl sufoxide (DMSO) and subsequently diluted in FBS-free medium to a final DMSO concentration of 0.5%. Therapeutic exposure parameters (total *c*_max_ values) were obtained from the literature (Supplementary Table 1) and PHH and animal spheroids were exposed to 1×, 5×, and 20× of the respective concentrations. Starting on days 7 or 6 for PHH and animal spheroids, respectively, cells were treated with freshly prepared exposure medium every 48–72 h for a total of 14 days (Figure 1). This 2-week experimental time frame was chosen based on our previous data that indicated that sensitivity towards hepatotoxic compounds increases with prolonged exposure time (Bell *et al.*, 2016, 2017). On the last day, viability was assessed by ATP quantifications using the CellTiter-Glo Luminescent Cell Viability Assay (Promega, Sweden). Luminescence signals were measured using a MicroBeta2 LumiJET Microplate Reader (PerkinElmer, USA).

##### Statistical analysis and hepatotoxicity predictions

Viability measurements for each compound were normalized to the corresponding DMSO control on the same plate. Statistically significant differences in viability were defined on the basis of heteroscedastic 2-tailed *t*-tests between control and treatment samples (mostly 6–8 spheroids per condition) using *p* < .05 as a threshold. Compounds were experimentally classified as hepatotoxic if they resulted in a statistically significant reduction of viability below 80% of control levels. True positive (TP) and false negative (FN) compounds are drugs that are implicated in DILI *in vivo* in man and are predicted based on *in vitro* data to be hepatotoxic or nonhepatotoxic, respectively. Conversely, true negatives (TNs) and false positives (FPs) are compounds that do not cause DILI in the clinics and are experimentally predicted to be nonhepatotoxic or hepatotoxic, respectively. Sensitivity is defined as $\frac{\sum TP}{\sum TP+\sum FN}$ and specificity as $\frac{\sum TN}{\sum TN+\sum FP}$. The positive predictive values are calculated as $\frac{\sum TP}{\sum TP+\sum FP}$ and the negative predictive values as $\frac{\sum TN}{\sum TN+\sum FN}$. The overall predictive accuracy is defined as $\frac{\sum TP+\sum TN}{\sum TP+\sum TN\sum FP+\sum FN}$.

## RESULTS

### 

#### Compound Selection and DILI Classification

**Figure 2.**
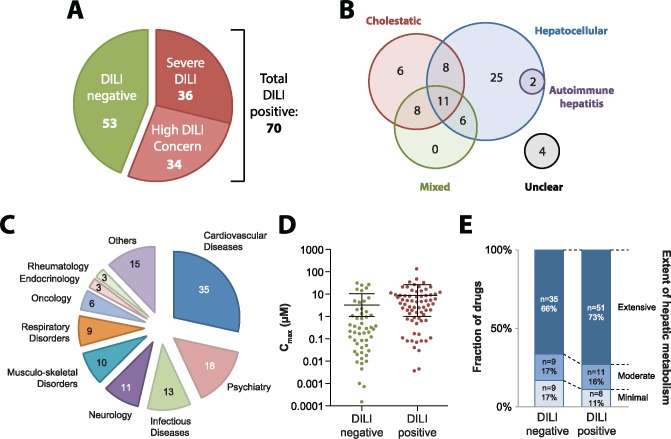
Overview of the compound panel tested in this study. A, Pie chart showing the classification of 123 compounds into 70 that have been associated with DILI events in the clinic (shades of red) and 53 that have not been reported to cause clinically apparent liver injury (green). B, Venn-diagram visualizing the reported DILI patterns for the 70 DILI positive compounds. C, Pie chart depicting the distribution of the 123 tested compounds across therapeutic areas. D, Therapeutic *c*_max_ serum concentrations of DILI positive and DILI negative medications are overall similar. E, Most drugs tested in this study are extensively metabolized by the liver. Overall, the DILI negative compounds show a slight tendency for less hepatic metabolism compared with DILI positive drugs.

To assess the predictive power of CD-spheroids to accurately detect the hepatotoxicity of drug candidates, we first selected an extensive panel of 123 drugs with high-quality data about their hepatotoxic liability in the clinics. DILI classification of compounds was based on regulatory classifications and available expert assessments (Chen *et al.*, 2011; Guo *et al.*, 2005; O’Brien *et al.*, 2006; Sakatis *et al.*, 2012; Suzuki *et al.*, 2010; Xu *et al.*, 2008) (Supplementary Table 2). Overall, our test panel consisted of 53 drugs that were not linked to clinically apparent liver injury and 70 compounds that showed direct associations to human hepatotoxicity events (Figure 2A). Depending on whether or not these DILI positive agents were previously withdrawn from the market or received black box warnings due to hepatotoxicity, caused acute liver failure, or resulted in hepatic necrosis, they were further classified into drugs causing severe DILI (*n* = 36) and compounds with DILI concern (*n* = 34), respectively.

The DILI positive compounds used here have been reported to provoke diverse patterns of liver damage with 74% and 47% of compounds being implicated in hepatocellular (*n* = 52) and cholestatic injuries (*n* = 33), respectively (Figure 2B). Furthermore, 2 compounds (diclofenac and nitrofurantoin) were also associated with cases of idiopathic autoimmune hepatitis (Björnsson *et al.*, 2010; Scully *et al.*, 1993). The tested compounds were from diverse therapeutic areas with cardiovascular diseases, psychiatry, and infectious diseases being overall most abundantly represented (Figure 2C). Notably, therapeutic exposure levels of DILI negative drugs (*c*_max_ = 3.2 ± 7.3 SD) were slightly lower than of DILI positive compounds (*c*_max_ = 8.8 ± 18 SD; *p* = .04; Figure 2D). Most drugs in our test panel are extensively metabolized in the liver (*n* = 86, Figure 2E and Supplementary Table 2). Among DILI negative drugs, slightly fewer compounds have at least moderate hepatic metabolism (83%) compared with drugs associated with clinical DILI events (89%).

#### CD-Spheroids Predict Drug-Induced Hepatotoxicity

**Figure 3.**
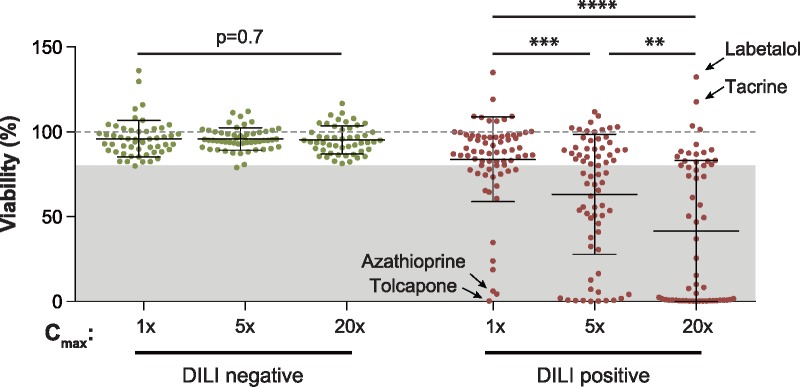
Compounds implicated in clinical DILI events exhibit substantial hepatotoxicity in the CD-spheroid system. Overview of the experimentally determined viabilities for DILI negative (green) and DILI positive (red) compounds at 1×, 5×, and 20× of the therapeutic serum concentration (*c*_max_) after 2 weeks of exposure. Viability as determined by ATP quantifications relative to untreated controls is shown. The dashed line indicates viability of the respective control spheroids (100%). The gray shaded box highlights the viability interval in which compounds are classified as hepatotoxic (<80%). Note that viability decreases dose-dependently when hepatocytes are exposed to DILI positive but not to DILI negative compounds. Error bars indicate SD **, ***, and **** indicate *p* < .01, *p* < .001, and *p* < .0001 in a 2-tailed heteroscedastic *t*-test, respectively.

Next, we evaluated the hepatotoxicity of all given compounds in a repeated exposure setting to accurately mimic human DILI events. We assessed hepatotoxicity at 1×, 5×, and 20× of the therapeutic concentrations detected in patient serum (*c*_max_). Exposure of PHH spheroids to drugs without DILI liability did not significantly reduce hepatocyte viability at any concentration tested (average viability of 96% at all 3 exposure levels; *p* > .7). In contrast, we found that exposure to DILI positive compounds resulted in an overall dose-dependent decrease in viability (Figure 3). Of the DILI positive compounds, the COMT inhibitor tolcapone, the chemotherapeutic agent azathioprine and the withdrawn antifungal ketoconazole were found to be overall most hepatotoxic and exerted substantial cytotoxicity (viability <10%) already at therapeutic exposure levels. In contrast, the α- and β-adrenergic antagonist labetalol, the cholinesterase inhibitor tacrine and the antifungal griseofulvin did not provoke hepatotoxicity even at the highest concentration tested (viability >100%).

**Figure 4.**
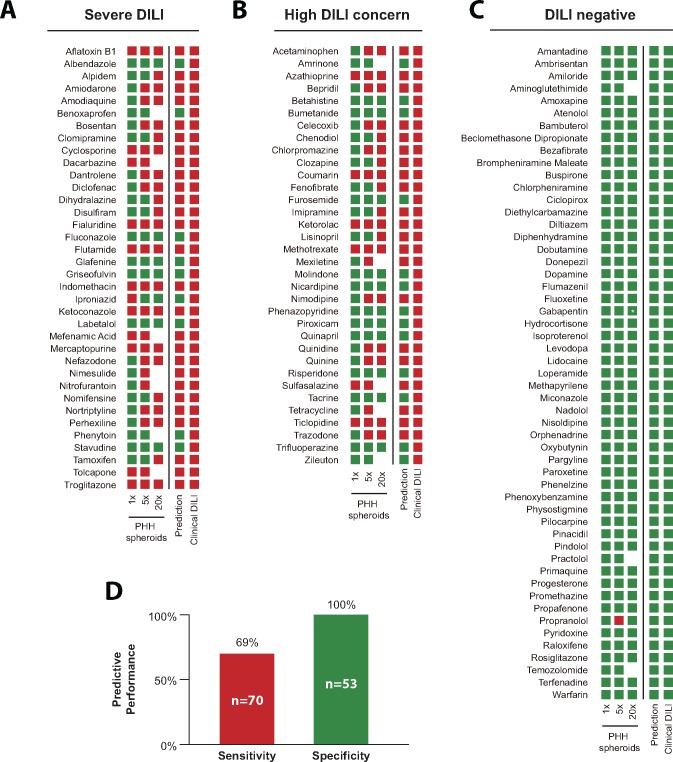
The CD-spheroid model faithfully flags compounds with hepatotoxic liabilities. Results of experimental hepatotoxicity assessments are shown for compounds causing severe DILI (*n* = 36; A) for drugs with DILI concern (*n* = 34; B) and for DILI negative compounds (*n* = 53; C). Red boxes indicate that the average hepatocyte viability was decreased to <80% of the respective controls with statistical significance (*p* < .05, mostly *n* = 6–8 biological replicates per condition). If these conditions are not met, the evaluation is classified negative (green). Note that drugs for which toxicity was indicated at low concentrations followed by nontoxic evaluations at higher concentrations were predicted to be DILI negative (iproniazid and propranolol). D, Overall, the spheroid model flagged 69% of DILI positive compounds as hepatotoxic without any FP results (specificity 100%). * Note that the highest exposure concentration for gabapentin was 17.3× *c*_max_.

Of the 70 compounds with DILI liabilities in humans, 48 were successfully predicted to be hepatotoxic, as defined by a statistically significant reduction of viability below 80% of control levels, resulting in a sensitivity of 69% and a negative predictive value of 71% (Figs. 4A–C). Importantly, none of the 53 DILI negative compounds were predicted to be hepatotoxic, resulting in a specificity and positive predictive value of 100% (Figure 4D).

#### CD-Spheroids Distinguish Between Toxic and Nontoxic Structural Analogues

**Figure 5.**
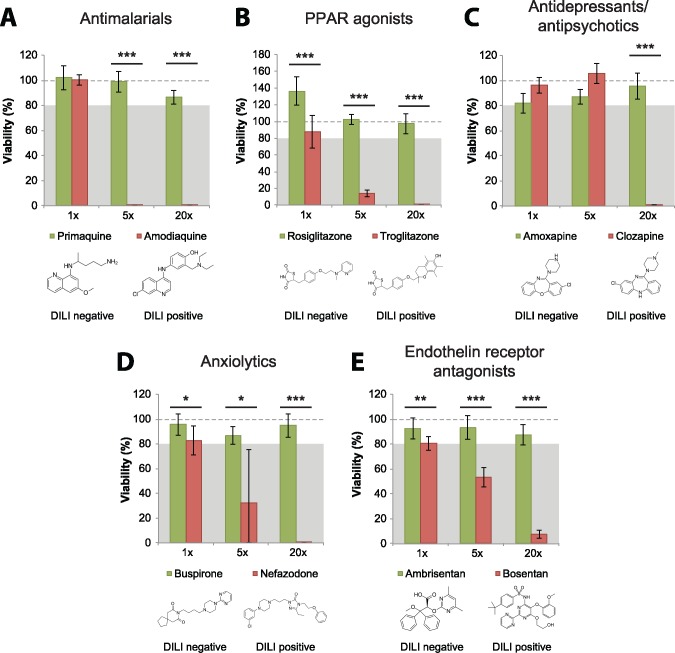
The CD-spheroid model can distinguish between hepatotoxic and nonhepatotoxic structural analogues. Hepatotoxicity of the structurally related antimalarials primaquine (DILI negative) and amodiaquine (DILI positive; A), the antidiabetic PPAR agonists rosiglitazone (DILI negative) and troglitazone (DILI positive; B), the antidepressant amoxapine (DILI negative) and the antipsychotic clozapine (DILI positive; C), the anxiolytics buspirone (DILI negative) and nefazodone (DILI positive; D), as well as the endothelin receptor antagonists ambrisentan (DILI negative) and bosentan (DILI positive; E) were compared in 3D PHH spheroids. Note that all 5 DILI positive compounds were clearly identified as hepatotoxic, whereas their DILI negative analogues revealed no toxic liability. The dashed line indicates viability of respective control spheroids (100%). The gray shaded boxes highlight the viability interval in which compounds are classified as hepatotoxic (<80%). *, **, and *** indicate *p* < .05, *p* < .01, and *p* < .001 in a 2-tailed heteroscedastic *t*-test, respectively.

To further substantiate the sensitivity and specificity assessment of the assay, we evaluated the capabilities to distinguish between DILI positive and negative structural analogues. Primaquine and amodiaquine are structurally related compounds for malaria prophylaxis and therapy. Although primaquine has not been linked to clinically apparent liver injury in over 50 years of use, amodiaquine is estimated to cause severe hepatic ADRs in 1 out of 15, 650 patients and has been directly linked to fatal cases of DILI (Markham *et al.*, 2007; Taylor and White, 2004). In our CD-spheroid system, primaquine did not cause apparent toxicity at any concentration tested, whereas amodiaquine was highly toxic at 5× and 20× of therapeutic serum levels (Figure 5A). Moreover, toxicity of the antidiabetic PPARγ agonist troglitazone, which has been withdrawn from the market due to its association with idiosyncratic DILI and liver failures in around 1 in 10 000-treated patients (Kohlroser *et al.*, 2000), was clearly detected, whereas its nonhepatotoxic structural analogue rosiglitazone did not indicate any hepatic liabilities (Figure 5B).

Although the antipsychotic clozapine is associated with elevated liver enzyme levels in around 30% of patients and can cause fulminant liver failure (Chang *et al.*, 2009; Wu Chou *et al.*, 2014), the structurally related antidepressant amoxapine has not been reported to cause liver injury. In agreement with these clinical reports, amoxapine was not found to be toxic at any concentration tested, whereas clozapine caused severe liver toxicity (Figure 5C). Similarly, the DILI negative anxiolytic buspirone was not hepatotoxic, whereas nefazodone, a related compound withdrawn from the market due to severe hepatotoxicity, provoked severe toxicity at 5× and 20× of the therapeutic exposure levels (Figure 5D;Choi, 2003).

In addition, we tested the structurally related endothelin receptor inhibitors ambrisentan and bosentan (Figure 5E). Bosentan use mandates monthly liver function tests (LFTs) and has been repeatedly implicated in acute and severe liver injury (Humbert *et al.*, 2007). The mechanism behind its toxicity is believed to be inhibition of the bile salt export pump (BSEP) resulting in reduced biliary efflux (Fattinger *et al.*, 2001; Kenna *et al.*, 2015). In contrast, ambrisentan has not been implicated in impaired bile flux or hepatotoxicity *in vitro* or *in vivo* (Fattinger *et al.*, 2001; Kenna *et al.*, 2015), even in patients who previously discontinued bosentan due to LFT abnormalities (McGoon et al., 2009). Here, we clearly detected bosentan-induced liver injury, whereas the ambrisentan exposure did not result in a significant reduction of hepatocyte viability. Thus, we find that the CD-spheroid platform was able to accurately distinguish between hepatotoxic and nonhepatotoxic structurally related drugs, underlining its utility for the evaluation of drug candidates at chemical derivatization stages in drug development.

#### Predictive Performance of Primary Hepatocyte Spheroids From Preclinically Important Animal Models

**Figure 6.**
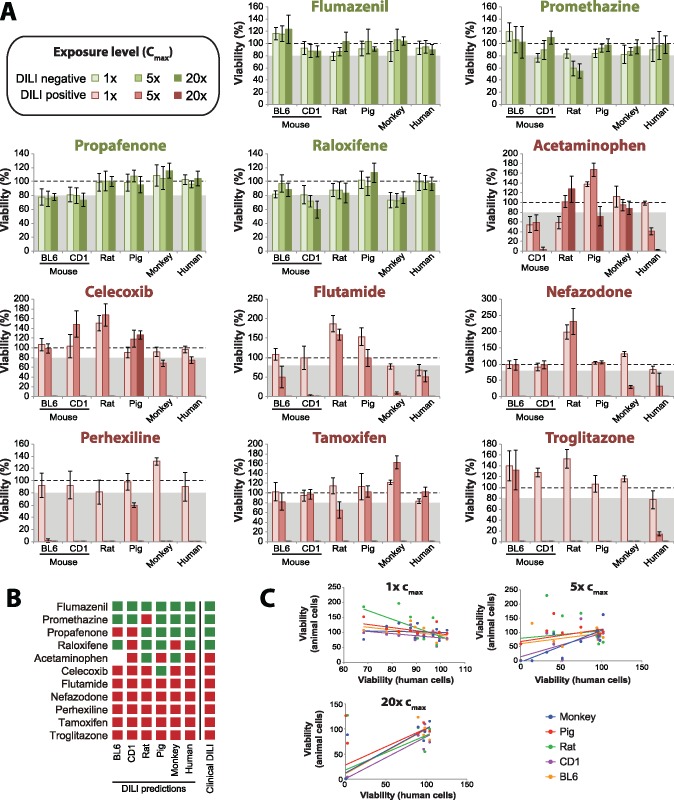
Cross-comparison of spheroids generated from primary hepatocytes of important preclinical animal model species. A, Viability quantifications of spheroids established from primary hepatocytes from BL6 and CD1 mice, as well as from Wistar rat, minipig, rhesus monkey, and human after 2 weeks of exposure are shown (*n* = 6–8 biological replicates per condition). Different shades of green and red indicate exposure to 1×, 5×, and 20× the therapeutic exposure levels (*c*_max_) in humans for DILI negative and DILI positive compounds, respectively. The dashed line indicates viability of the respective species controls (100%). The grey shaded boxes highlight the viability interval in which compounds are classified as hepatotoxic (<80%). Error bars indicate SD. B, Based on data shown in A, predictions about the toxic liability of compounds across species are shown. Red and green boxes indicate hepatotoxic and nonhepatotoxic predictions, respectively. C, Scatter plots depicting the concordance between human (abscissa) and animal (ordinate) viability quantifications for each of the 11 tested compounds. Note that while the concordance between toxicity evaluations across species are overall similar at the highest (20×) exposure concentration, toxicity predictions at low (1×), and intermediate (5×) concentrations are highly discrepant, suggesting substantial variability in thresholds at which hepatotoxicity manifests across species.

Next, we evaluated the potential of our spheroid model to replicate inter-species differences in hepatotoxicity. To this end, we generated 3D spheroids of primary hepatocytes from 2 common mouse strains (inbred C57BL/6 and outbred CD1), as well as from Wistar rat, minipig and rhesus monkey, and compared the hepatotoxicity signals of 11 selected drugs (4 DILI negative, 7 DILI positive). Of the 4 compounds without clinical DILI implications, propafenone (C57BL/6 and CD1) and raloxifene (CD1 only) were incorrectly flagged as hepatotoxic when using mouse spheroids (Figs. 6A and 6B). Raloxifene moreover resulted in mild but significant toxicity in rhesus monkey spheroids and spheroids of rat cells indicated toxicity of promethazine.

Of the 7 DILI positive drugs, 6 (flutamide, nefazodone, perhexiline, tamoxifen, and troglitazone) were flagged as hepatotoxic by primary hepatocyte spheroids from all preclinical model species analyzed, albeit at variable threshold levels. In contrast, the detection of acetaminophen toxicity was species-specific. Although the dominant fraction (around 90%) of acetaminophen is inactivated by sulfation or glucuronidation, 5%–10% are metabolically activated by CYP2E1 and CYP3A4 in the human liver to the reactive metabolite *N*-acetyl-p-benzoquinone imine (NAPQI) that exerts its hepatotoxicity by forming protein adducts (Yoon *et al.*, 2016). Although the metabolism of acetaminophen is similar between mice, rats, and humans, mitochondrial protein adducts have only been observed in mice and humans, whereas rats are resistant to acetaminophen-induced hepatotoxicity (McGill *et al.*, 2012b). In agreement with these *in vivo* data, acetaminophen toxicity was detected in mouse but not in rat spheroids (Figs. 6A and 6B). Interestingly, also 3D-cultured monkey hepatocytes were refractory to acetaminophen hepatotoxicity while hepatotoxicity was detected in human spheroids, indicating that even hepatocytes from relatively closely related species can differ substantially in toxicity signatures.

Although spheroids from minipig could overall reliably distinguish between the hepatotoxic and nonhepatotoxic drugs in our test panel, they showed no indication of hepatotoxicity to the COX2 inhibitor celecoxib (viability >100%), whereas this drug was highly toxic in spheroids from both mouse strains, rat, monkey and human (viability for all models <2% of respective controls at 20× *c*_max_ exposure levels; Figure 6A). In summary, while the DILI predictions based on spheroids from human and animal hepatocytes were overall similar at relatively high concentrations (20× *c*_max_), the concordance between toxicity thresholds differed substantially across species (Figure 6C). At low (1× *c*_max_) and intermediate (5× *c*_max_) exposure levels, prediction results were highly discrepant with spheroids from monkey being the most similar to human cultures (*R*^2^ = 0.66). These results indicate that inter-species differences in sensitivity are evident even in isolated hepatic systems that allow to abstract from systemic absorption, distribution, and excretion phenomena.

## DISCUSSION

Although toxicity tests in animal models constitute indispensable regulatory requirements, inter-species differences in drug absorption, distribution, metabolism, and excretion necessitate the complementation of animal experiments with hepatic *in vitro* models based on human cells. As a consequence of these species differences, some candidate drugs that are hepatotoxic in man but not in animal systems are taken forward into the clinics with sometimes catastrophic outcomes for drug developers and trial participants. One example for such a compound is fialuridine, a nucleoside analog developed for the treatment of viral hepatitis, which did not show indications of hepatotoxicity in any preclinical species, including rat, mouse, dog, and cynomolgus monkey (Manning and Swartz, 1995). Despite this negative risk assessment, 7 out of 15 volunteers in a phase I clinical trial of fialuridine exhibited lactic acidosis and acute liver failure few weeks after commencing therapy, 5 of whom died (McKenzie *et al.*, 1995). Importantly, PHH spheroids constitute to our knowledge the only *in vitro* system to date that has been shown to successfully detect fialuridine hepatotoxicity at clinically relevant concentrations, thus demonstrating the potential of this model for the reduction of FN toxicity predictions. Overall, our platform correctly identified 69% of drugs with hepatotoxic liabilities, thereby showing a substantially higher sensitivity than previously reported systems (Table 1).

**Table 1.** Comparison of the Predictive Performance of the Presented Spheroid Platform With Previously Published PHH Systems

	2Dsw	MPCC	Spheroid models		
References	Xu *et al.* (2008)	Khetani *et al.* (2013)	Proctor *et al.* (2017)		This study
Exposure time	24 h	9 days	14 days		14 days
Tested compounds (DILI positive/DILI negative)	344 (200/144)	45 (35/10)	110 (69/41)		123 (70/53)
Fraction DILI positive	**0.58**	**0.78**	**0.63**		**0.57**
*C*_max_	**100×**	**100×**	**25×**	**100×**	**20×**
**Statistical Metric**					
TPs	101	23	33	41	48
FNs	99	12	36	28	22
TNs	144	9	38	33	53
FPs	0	1	3	8	0
Sensitivity	51%	66%	48%	59%	69%
Specificity	100%	90%	93%	80%	100%
Positive predictive value	100%	96%	92%	84%	100%
Negative predictive value	59%	43%	51%	54%	71%
Accuracy	71%	71%	65%	67%	82%

2Dsw, 2D sandwich culture; MPCC, micropatterned cocultures.

In addition to FN prediction, species differences can result in the attrition of nontoxic compounds due to toxicity signals that are specific to the employed animal model. Here, we demonstrate that the spheroid model presented in this study is compatible with the culture of primary hepatocytes from commonly used preclinical rodent (mouse and rat) and nonrodent (minipig and rhesus monkey) model species. Importantly, species-specific toxicity patterns of acetaminophen, which exerted hepatotoxic effects in mouse but not in rat hepatocytes, were recapitulated in our assay, in agreement with previous reports (McGill *et al.*, 2012a). As the regulatory requirement commonly mandates the use of at least 1 rodent and 1 nonrodent model, there is flexibility regarding the exact choice of model system. Our data indicate that comparative cross-species *in vitro* evaluations using 3D spheroids can assist in the selection of the preclinical model species whose toxicity profiles for the given compound are expected to most closely resemble human toxicity. Thereby, these experiments are expected to reduce the risks of attrition of promising drug candidates due to animal-specific hepatotoxicity events and raise success rates in preclinical development.

In recent years, multiple culture paradigms have been presented in which PHH retain their molecular phenotypes and hepatic functions for extended periods of time (up to 5 weeks), which constitutes an integral feature for the accurate modeling of human DILI. Xu *et al.* evaluated the acute hepatotoxicity of 344 compounds (200 DILI positives and 144 DILI negatives) in 2D sandwich cultures of PHH using high content cellular imaging (Xu *et al.*, 2008). Although their platform did not result in FPs, they only detected hepatotoxicity of 101 out of 200 compounds (sensitivity = 51%). Of the 99 compounds whose liver toxicity was not recognized in their study, 23 overlapped with our test set. Notably, we successfully detected 10 of these 23 compounds (43%) as hepatotoxic, including imipramine, clomipramine, flutamide and lisinopril (Supplementary Table 3), despite using 5-fold lower exposure levels (20× *c*_max_ instead of 100×). In contrast, only 7 of 29 compounds (24%) were flagged as hepatotoxic at high exposure levels in sandwich culture, but were predicted to be DILI negative in CD-spheroids. Moreover, 4 of these 7 drugs (albendazole, labetalol, trifluoperazine, and zileuton) were associated with only slight increases in reactive oxygen species levels (Xu *et al.*, 2008), which might not compromise cell viability.

Besides screens in sandwich cultures, 1 hepatotoxicity evaluation using 45 compounds (35 DILI positives and 10 DILI negatives) has been published in micropatterned cocultures (MPCC) (Khetani *et al.*, 2013). In this platform, hepatocytes are cultured on predefined collagen-coated domains and surrounded by murine embryonic fibroblasts. The authors evaluated toxicity towards a repeated dosing regimen using the same concentrations (100×) as Xu *et al.* Of the 35 DILI positive drugs, 11 overlapped with our testing panel and both assays yielded identical prediction results, emphasizing the maintenance of relevant hepatic phenotypes in the MPCC system. Of the 10 DILI negative compounds in their study, 7 were assessed here in CD-spheroids. In MPCC, 6 of these compounds were classified as negative, whereas lidocaine was falsely flagged as hepatotoxic. In contrast, in our screen none of the 7 DILI negative compounds produced indications of hepatotoxicity.

In a recent study, Proctor *et al.* predicted the hepatotoxic liabilities of 110 drugs in PHH spheroids formed using a hanging-drop method and exposure to 100× *c*_max_ concentrations for 2 weeks in undisclosed media formulations (Proctor *et al.*, 2017). They reported an overall sensitivity of 59% (41/69 DILI positives) and a specificity of 80% (33/41 DILI negatives). 28 compounds of their drug panel with clinical DILI indications were evaluated in our study. Of these, all 19 compounds that were classified as positives by Proctor *et al.* were also identified as hepatotoxic in our PHH spheroid screen. In addition, we revealed the DILI liability of 3 out of the 9 compounds (dantrolene, indomethacin, and methotrexate) that were previously missed despite using 5-fold lower exposure levels in our study. Importantly, the superior sensitivity in our system was not compromised by reduced specificity. Of 10 overlapping drugs without clinical DILI signals, 2 (rosiglitazone and fluoxetine) were flagged as hepatotoxic by Proctor *et al.*, whereas none of the 10 were falsely classified as DILI positive in our screen. The reduced sensitivity and specificity in the study by Proctor *et al.* potentially stems from differences at the level of molecular phenotypes, e.g. due to MAPK-mediated downregulation of CYPs by growth factors (Murray *et al.*, 2010). However, as media conditions of their model are not disclosed, the underlying causes can only be speculated about.

Notably, the hepatotoxicity of indomethacin and methotrexate was clearly detected already at 1× *c*_max_ exposure levels in the CD-spheroid system, whereas none of the previous studies detected these toxicity risks even at 100-fold higher concentrations. Indomethacin toxicity is suggested to involve a toxic intermediate whereas acute methotrexate toxicity is believed to originate from oxidative stress due to elevated homocysteine levels caused by an indirect inhibition of methylenetetrahydrofolate reductase, the enzyme catalyzing the biosynthesis of methionine from homocysteine (Aithal, 2011). Although the presented CD-spheroid system exhibited a higher sensitivity than previous models, 31% of hepatotoxic compounds could not be detected using the given concentrations. One reason for these discrepancies could be extensive first pass metabolism, which entails that hepatocytes *in vivo* are exposed to substantially higher concentrations than measured in plasma, which we used as a basis for our choice of exposure levels. Examples for FN drugs with extensive first pass metabolism include tacrine (Madden *et al.*, 1995), risperidone (El-Hage and Surguladze, 2010), and albendazole (Rawden *et al.*, 2000; Villaverde *et al.*, 1995). Thus, direct measurements of hepatic exposure concentrations are expected to improve the selection of exposure concentrations, particularly for drugs with substantial first-pass metabolism. In contrast, evaluating more donors is not expected to substantially increase assay performance, as previous studies found only minor inter-individual differences in the susceptibility to hepatic toxicity between donors (Sison-Young *et al.*, 2017).

In conclusion, the CD-spheroid system presented in this work exceeded both the sensitivity and the specificity of all previously published *in vitro* assays at substantially lower exposure levels (Table 1). Furthermore, we showed that these improved results are not due to the testing of different drugs, but rather that the predictive performance was also substantially higher when only overlapping subsets of compounds were compared.

## SUPPLEMENTARY DATA

Supplementary data are available at *Toxicological Sciences* online.

## Supplementary Material

Supplementary Data
